# New Substituted 5-Benzylideno-2-Adamantylthiazol[3,2-b][1,2,4]Triazol-6(5*H*)ones as Possible Anti-Inflammatory Agents

**DOI:** 10.3390/molecules26030659

**Published:** 2021-01-27

**Authors:** Christophe Tratrat, Michelyne Haroun, Aliki Paparisva, Charalmpos Kamoutsis, Anthi Petrou, Antonis Gavalas, Phaedra Eleftheriou, Athina Geronikaki, Katharigatta N. Venugopala, Hafedh Kochkar, Anroop B. Nair

**Affiliations:** 1 Department of Pharmaceutical Sciences, College of Clinical Pharmacy, King Faisal University, Al-Ahsa 31982, Saudi Arabia; mharoun@kfu.edu.sa (M.H.); or kvenugopala@kfu.edu.sa (K.N.V.); anair@kfu.edu.sa (A.B.N.); 2 School of Pharmacy, University of Thessaloniki, 54124 Thessaloniki, Greece; aliki-@hotmail.com (A.P.); anthi.petrou.thessaloniki1@gmail.com (A.P.); agavalas@pharm.auth.gr (A.G.); 3 School of Pharmacy, University of Patras, 26504 Patra, Greece; kamoutsi@upatras.gr; 4 Department of Biomedical Sciences, School of Health Sciences, International Hellenic University, 57400 Thessaloniki, Greece; elfther@ihu.gr; 5 Department of Biotechnology and Food Technology, Durban University of Technology, Durban 4001, South Africa; 6 Department of Chemistry, College of Science, Imam Abdulrahman Bin Faisal University, Dammam 31441, Saudi Arabia; hbkochkar@iau.edu.sa; 7 Basic & Applied Scientific Research Center, Imam Abdulrahman Bin Faisal University, Dammam 31441, Saudi Arabia

**Keywords:** anti-inflammatory, thiazole, triazole, COX, LOX, docking, carrageenan, NDGA

## Abstract

Background: Inflammation is a complex response to noxious stimuli promoted by the release of chemical mediators from the damaged cells. Metabolic products of arachidonic acid, produced by the action of cyclooxygenase and lipoxygenase, play important roles in this process. Several non-steroidal anti-inflammatory drugs act as cyclooxygenase inhibitors. However, almost all of them have undesired side effects. Methods: Prediction of the anti-inflammatory action of the compounds was performed using PASS Program. The anti-inflammatory activity was evaluated by the carrageenan paw edema test. COX and LOX inhibitory actions were tested using ovine COX-1, human recombinant COX-2 and soybean LOX-1, respectively. Docking analysis was performed using Autodock. Results: All designed derivatives had good prediction results according to PASS and were synthesized and experimentally evaluated. The compounds exhibited in vivo anti-inflammatory action with eleven being equal or better than indomethacin. Although, some of them had no or low inhibitory effect on COX-1/2 or LOX, certain compounds exhibited COX-1 inhibition much higher than naproxen and COX-2 inhibition, well explained by Docking analysis. Conclusions: A number of compounds with good anti-inflammatory action were obtained. Although, some exhibited remarkable COX inhibitory action this activity did not follow the anti-inflammatory results, indicating the implication of other mechanisms.

## 1. Introduction

Inflammation is defined as the reaction to (a) invasion of an infectious agent, (b) a challenge by an antigen, or (c) even a simple physical injury [1,2]. Inflammation is a complex response adjustment, which is activated by noxious stimuli and conditions, such as disease and/or injury of a tissue and is an important non-specific defense mechanism. Inflammation is triggered and promoted by the release of chemical mediators from the damaged tissue and migrating cells.

Although, the specific chemical mediators may vary according to the type of the inflammatory process, histamine and bradykinin secretion by mast cells are among the first events of inflammation [3]. Several other mediators are also released among which neurotransmitters such as the substance P (SP) [4] of the tachykinin family and the calcitonin gene-related peptide (CGRP) which may be involved in vasodilation and increased vascular permeability. In addition, a number of pro-inflammatory compounds, such as tumor necrosis factor alpha (TNF-α) and interleukin-1 beta (IL-1β) are over expressed while prostaglandins and leukotrienes [5,6], also involved in vascular properties modulation, produced by the action of COX (mainly COX-2) and LOX enzymes on the arachidonic acid released by phospholipase A2 (PLA2) which is also activated.

Various non-steroidal anti-inflammatory drugs (NSAIDs) are broadly used clinically as inhibitors of cyclooxygenase (COX) isoenzymes COX-1 and COX-2, the isoenzyme induced during inflammation. Unfortunately, almost all of them have several undesired side effects. Moreover, dual acting COX/LOX inhibitors are under investigation, while several other molecules of the inflammation mechanism have been proposed as drug targets. Interestingly, recent research has added a novel mechanism of action, as CGRP release inhibitor, to the approved NSAID nimesulide which is known to possess COX inhibitory action, explaining its role in migraine treatment [7].

Thiazole ring is an important heterocyclic system possessing a wide range of pharmacological activities. Among them are bacteriostatics, antibiotics [8,9,10], local anesthetics [11], anti-inflammatory [12,13,14,15], analgesic and antipyretics [16], anti-HIV [17,18], antiallergic [19], antihypertensives [20] against schizophrenia [21] and hypnotics [22]. Furthermore, some drugs, such as meloxicam, a new NSAID [23], the sulfathiazole, simple sulfonamide antibacterial, as well as niridazole, stronger medicine drug against schistosomiasis [24] contain thiazole ring in its molecules.

Another heterocyclic ring possessing numerous types of biological activities is thiazolidinone. Among them are anti-inflammatory [25,26,27], antimicrobial [28,29], anticancer [30,31], antidiabetic [32], anti-HIV [33].

Except of thiazole and thiazolidinone rings 1,2,4-triazole system attracted scientific interest due to the anti-inflammatory [34,35], antibacterial [36,37], anticancer [38], antiviral [38] and many other activities [39,40,41].

Thus, the thiazolo [3,2-b]1,2,4-triazoles are compounds with broad spectrum of biological activities such as antimicrobial [42], anti-inflammatory [43] and analgesic [43] anticancer [44]. In addition, some adamantane derivatives except of their well-known antiviral activity against influenza [45,46] and HIV viruses [47] also exhibit antimicrobial [48], anti-inflammatory activities [49].

These findings prompted us to incorporate thiazolo [3,2-b]1,2,4-triazole with adamantine ring in one frame in order to obtain compounds with improved/higher anti-inflammatory activity. PASS Program was used for computer aided prediction of the anti-inflammatory activity of the designed compounds before synthesis and experimental evaluation.

For a long time, patients have been cured with combination of drugs endowed with different medicinal activities. Computer-aided prediction of pharmacological activity spectra of compounds and drugs based on their structural formulas can be estimated by the drug design software PASS (Prediction of Activity Spectra for substances) [50,51,52] to perform investigations on anti-inflammatory agents acting as cyclooxygenase/lipoxygenase (COX/LOX) dual inhibitors. PASS is based on the robust analysis of structure-activity relationships in a heterogeneous training set, which includes 989,000 various chemical compound families endowed with different kinds of biological activities. The current version of PASS predicts more than 7900 types of biological activity including pharmacotherapeutic effects, mechanisms of action, interaction with drug-metabolizing enzymes, side effects, and toxicity [2,8]

## 2. Results and Discussion

### 2.1. Prediction of Anti-Inflammatory Activity of Designed Compounds

The anti-inflammatory activity of the twenty five substituted 5-benzylideno-2-adamantylthiazol[3,2-b][1,2,4]triazol-6(5*H*)ones previously synthesized [42] was predicted using the PASS (Prediction of Activity Spectra for substances) program (Table 1). Prediction was based on the comparison of structural characteristics of the compounds with that of known anti-inflammatory agents within a databank of 989,000 molecules. According to the results, all compounds exhibited a probability, Pa, to show anti-inflammatory action between 0.274 and 0.636, a high enough probability to encourage the experimental evaluation of the compounds.

### 2.2. In Vivo Inhibition of the Carrageenin-Induced Edema

The in vivo anti-inflammatory effect of the tested compounds was assessed by using the functional model of carrageenin-induced mouse paw edema and is presented as the percentage of inhibition of induction of edema at the right hind paw (Table 1).

Edema was measured by weight increase of the right hind paw in comparison to the uninfected left paw. Carrageenin-induced edema is a nonspecific inflammation that is highly sensitive to non-steroidal anti-inflammatory drugs (NSAIDs), and so carrageenin has been accepted as a suitable agent for the study of new compounds with anti-inflammatory activity.

Among the mediators involved in oedema formation during the first 3.5 h of carrageenin induced inflammation are histamine and bradykinin, the neuropeptides SP [4] and CGLP, prostaglandins [6], leukotrienes and reactive oxygen and nitrogen species [3]. The SP and CGLP peptides induce plasma extravasation through the NK1Rs and hyperemia through the calcitonin receptor-like receptor (CLR) of arterioles, respectively [53], while prostaglandin E (PGE2) induce vasodilation via the EP2/EP4 receptors [54]. In addition the Cysteinyl leukotriene LTC4 enhances vascular permeability through the action of CysLT1 receptor [53]. A CGRP receptor mediated mechanism has been indicated for the histamine action in rat paw inflammation, revealing a neuro-inflammatory process [55]. The production of molecules starts immediately after inflammatory triggering and reaches substantial levels during the first 3.5 h.

In conclusion, the test can reveal the activity of compounds with different anti-inflammatory mechanism including traditional COX inhibitors, LOX inhibitors or compounds acting through the CGRP-R or the tachykinin receptor processes.

Twenty-five thiazolo[3,2-b]1,2,4-triazole derivatives, previously synthesized [42] were evaluated for their anti-inflammatory activity, in vivo using groups of 6–10 mice of R’’’ strain. As shown in Table 1, the protection ranged up to 67%, while the reference drug, indomethacin, induced 47% protection at an equivalent dose. It was observed that compounds **5**, **8**, **11**, **15**–**19** and **22** showed good anti-inflammatory activity (51–67%) better than the reference drug indomethacin, while nine compounds (**1**, **4**, **6**, **9**, **10**, **12**, **14**, **20** and **21**) expressed activity almost equal to that of indomethacin (40–49%). The rest of compounds showed low activity.

The structure-activity relationships revealed that anti-inflammatory activity depends not only on the nature and the number of the substituents, but also on its position in benzene ring. Thus, introduction to the parent compound **1** a hydroxy group led to compounds with different activity. The activity of 3-OH and 4-OH derivatives (**2**, **3**) was almost equal, slightly less than that of compound **1**, while the presence of two hydroxyls in position 2,4 led to compound **4** with increased (49%) activity regarding parent compound **1** (40%). Introduction of a 3-OCH_3_ group to compound **3** increased the activity (compound **6**) to 46% compared to compounds **3** and **1**, while introduction of second methoxy group at position 5 (**7**) did not affect the activity of compound **3**. Shifting of hydroxyl group of compounds **6** from position 4 to position 2 furnished compound (**5**) with increased potency (58%). Replacement of 4-OH of compound **3** by a methoxy group resulted to compound (**8**) with very high activity (62%), while introduction of the second OCH_3_ group as well as third one in position 3 and 3,5 respectively decreased activity to 44% (**9**) and 49% (**10**) respectively. The presence of a dimethylamino substituent at position 4 (**11**) appeared to be favorable (55%). The influence of the nitro group was found to be dependent on its position. Thus, 2- and 4-NO_2_ derivatives (**12**, **14**) showed activity almost equal to that of parent compound **1**, while the presence of nitro group at position 3 (**13**) was the less potent compound (15%).

Among halogen derivatives, bromo derivatives, in general, appeared to be the most active. The order of activity for bromo derivatives can be presented as 2Br > 3-Br > 4-Br, whereas in case of fluoro (**15**, **16**) and chloro derivatives (**20**–**22**) substitution at position 4 appeared to be the most favorable. Introduction of the second chlorine (**23**–**25**) had negative effect on anti-inflammatory activity.

### 2.3. In Vitro Study of COX Inhibition

Derivatives of thiazole, 1,2,4-triazole and adamantane, according to the literature, have broad spectrum of biological activities, among which is inhibition of COX-1/COX-2 enzymes [56,57,58]. Taking this into account and in order to determine the effect of these compounds on the main pathways controlled by traditional NSAIDS evaluation of inhibitory activity on COX isoforms was performed. It was observed that inhibitory activity ranged between 0–93% (Table 2). As far as COX-1 inhibitory activity is concerned, two compounds (**3** and **8**) exhibited the same or higher activity than naproxen (60%). Among them the best activity was expressed by compound **3** which exhibited an IC_50_ of 1.28 μM, while compound **8** was the less active with IC_50_ 41.88 μM similar to that of naproxen (IC_50_ 40.1 µM Table 2). Replacement of 4-OH (**3**) with 4-OCH_3_ led to compound **8** with decreased inhibitory activity (60.5%). Insertion of the 3-OCH_3_ group (**6**) in compound **3** decreased threefold the activity, while introduction of the second methoxy group at position 5 (**7**) further diminished the inhibitory activity. The presence of halogens (F, Br) (**15**–**19**) seems to be unfavorable for inhibition of COX-1, while compound **23** (2,3-Cl) showed acceptable activity, but lower to that of naproxen.

As far as inhibition of COX-2 is concerned, only two compounds (**2** and **6**) exhibited the same activity with naproxen (65%). Removal of 3-OCH_3_ group of compounds **6**, as well as insertion of a second 5-OCH_3_ (**7**), significantly decreased the inhibition (Table 2). The replacement of 4-OH (**3**) with OCH_3_ group led to completely inactive compound (**8**). It is obvious that hydroxyl group is favorable feature for COX-1/COX-2 inhibitory activity, although a 3-OH group mostly favors COX-2 inhibition, while a 4-OH group the COX-1 inhibitory activity.

### 2.4. In Vitro Studies of LOX Inhibitory Activity

It is known that thiazole derivatives exhibit anti-inflammatory activity [7,8,9,10,11] and that in the process of inflammation different factors are involved. One of them is lipoxygenase enzyme. All this prompted us to study the mechanism of action of our compounds through this pathway, too. Thus, all compounds were tested for possible LOX inhibitory activity and results are presented in Table 2. From the results in the table it appears that the compounds do not show appreciable activity (5.7–31.4%). Accordingly, the conclusion that can be derived is that the inhibition of LOX enzyme is not responsible for the anti-inflammatory activity of tested compounds.

### 2.5. Docking Studies

**Figure 1:**
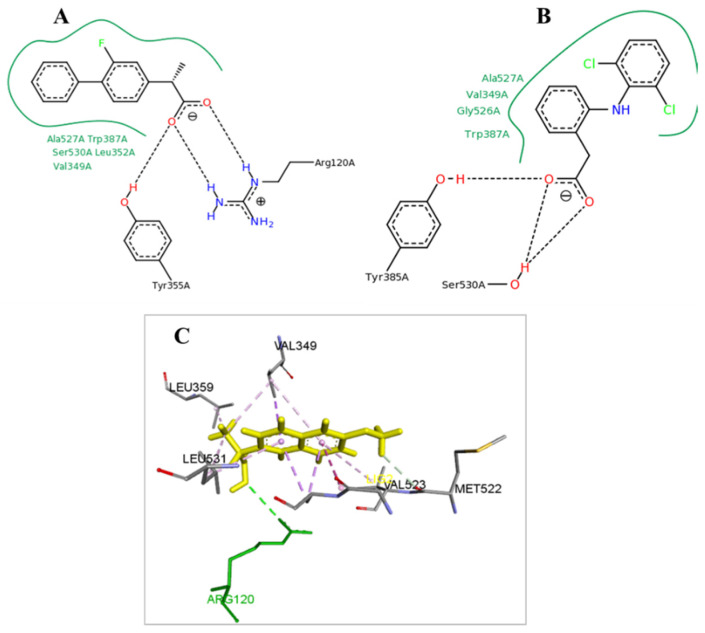
Binding of flubriprofen at active center of COX-1 (**A**) and diclofenac at active center of COX-2 (**B**). Binding of naproxen to COX-2 active center (**C**).

**Figure 2:**
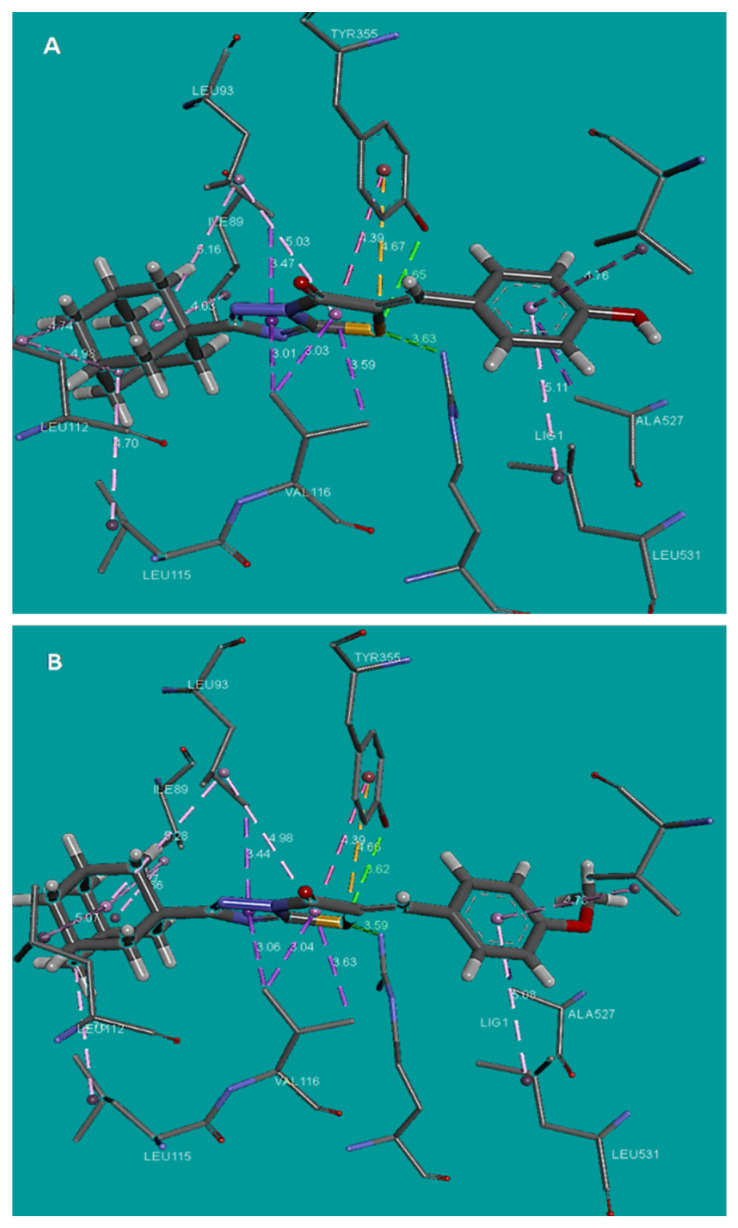
(**A**) Binding of compound **3** into the active site of COX–1, (**B**) Binding of compound **8** into the active site of COX–1.

Docking analysis was performed for two most active and the less active inhibitors of each one of COX isoenzymes (COX-1, COX-2). In both cases the estimated binding energy (Eest) was in accordance with activity of the compounds. Higher inhibitory action was predicted for compound **3** which showed the best binding affinity score and the lowest estimated binding energy (Eest = −8.2 Kcal/mol), the highest number of predicted hydrogen bonds and a mode of interaction with Arg120 and Tyr355 similar with that observed for flurbiprofen (Figure 1A and Figure 2A). More specifically a favorable H-bond is formed between the S atom of the thiazole ring and the side chain of Tyr355 (distance 3.56 Å) and another favorable H-bond between the S atom of the thiazole ring and the H of the side chain of Arg120 (distance 3.15 Å). It has been shown [54] that sulfur is close to oxygen in its hydrogen bond acceptor strength [55,56], forming weak to moderate hydrogen bonds with distances varying from 3.2–4.0 Å to 2.5–3.2 Å. The phenyl ring was found in proximity to the amino acids Leu531, Ala527, Val349 while the adamantine ring of the compound was found in the vicinity of the amino acids Ile89, Leu112 and Leu115 participating in various hydrophobic interactions. These results suggest that this compound docks well in the binding pocket and explains its high inhibitory action (Figure 2A).

The next best prediction was for compound **8** with good binding score and binding energy (Eest. = −7 Kcal/mol) and one hydrogen bond interaction with Tyr355 (Figure 2B) in which also the S atom of thiazole ring is involved.

On the other hand, the less active compound **18** did not participate in hydrogen bond formation with Arg120 and Tyr355 (Table 3) exhibiting increased estimated binding energy of −4.4Kcal/mol which explain the zero activity of the compound. In general, estimated biding energy lower than −5.5 Kcal/mol indicates extremely low up to zero, activity of a compound [59].

**Figure 3:**
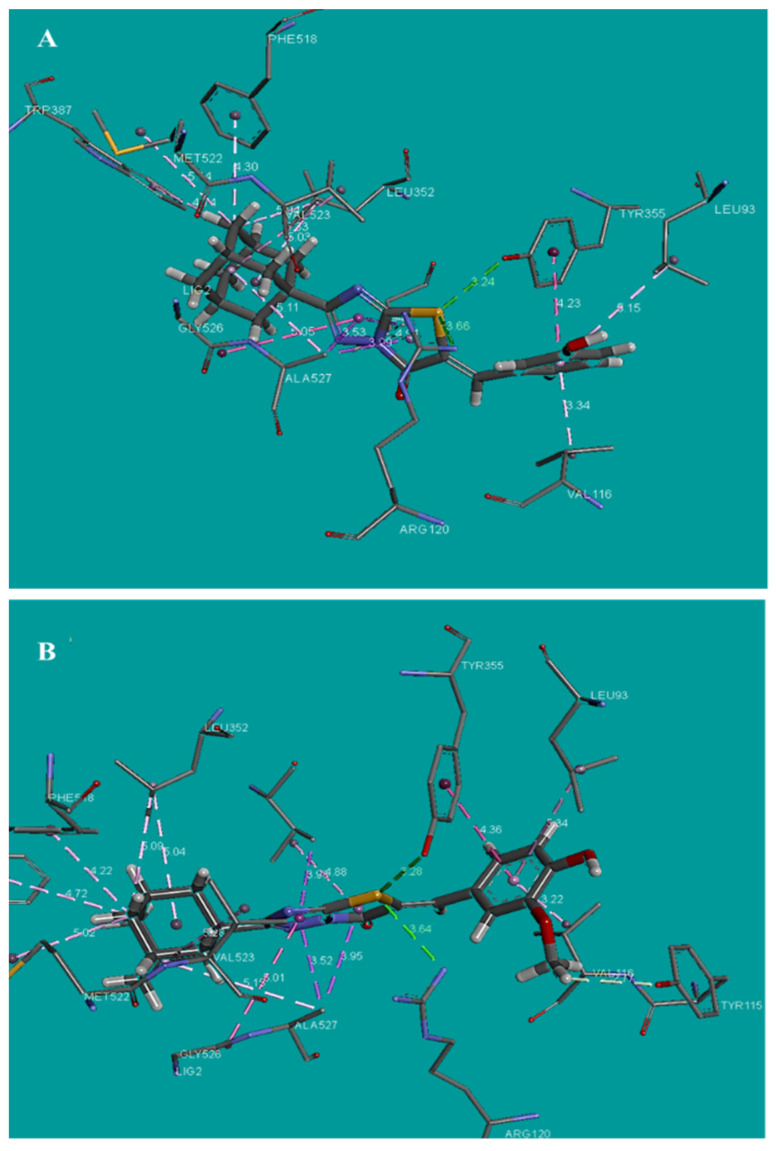
(**A**) Binding of **2** into the active site of COX–2, (**B**) Binding of **6** into the active site of COX–2.

Docking analysis of the compounds in COX-2 active site indicates Tyr355 and Arg120 involvement in hydrogen bond interactions with compounds **2** and **6**, and Arg120 participation in hydrogen bond interactions with the reference compound naproxen (Figure 1C). According to docking results, compound **2** showed the best predicted binding energy with the COX-2 isoenzyme. The next most promising compound according to predicted binding energy was compound **6** with similar predicted binding energy with naproxen (Figure 3A,B). Compound **16** showed high estimated binding energy and no hydrogen bond interactions (Table 4), which can explain the absence of inhibitory action. Compounds **2** and **6** dock well in the active site of COX-2 that consists of the amino acids Phe518, Ser353, Gly354, Ile517, Arg513, His90, Tyr355, and Arg120 (Figure 3A,B). The S of the thiazole ring forms two hydrogen bonds with Arg120 and Tyr355 of COX-2 (distance 3.24 Å and 3.66 Å and distance 3.28 Å and 3.64 Å for compounds **2** and **6** respectively), while the benzene ring settles in a hydrophobic cavity lined by Phe357, Tyr115, Leu359, Val116 and Met133, optimally oriented to make π-π interaction with the phenyl ring of Tyr355.

## 3. Materials and Methods

### 3.1. Chemistry

The synthesis of tested compounds was performed as described in our previous paper [42].

### 3.2. In Vivo Experiments. Inhibition of the Carrageenin-Induced Edema

The anti-inflammatory activity of the compounds synthesized was studied with the Carragenin-Induced Paw Edema acute inflammation test using an aqueous carrageenin solution (2%, 0,05 mL), [14] which was injected intradermally in the footpad of the right hind limb muscle (mice R’’’ groups of 6–10 animals), whereas the left end serves as a reference. In the experimental procedure both sex male mice were used, excluding pregnant. Animals were fasted during the time of the experiment. The tested compounds (0.01 mmol/kg body weight), were suspended in water, with few drops of Tween 80 and ground in a mortar before use and were given intraperitoneally simultaneously with the carrageenin injection.

### 3.3. COX Inhibitor Screening Assay in Vitro

The COX-1 and COX-2 activities of the compounds were measured using ovine COX-1 and human recombinant COX-2 enzymes included in the “COX I inhibitor Screening Assay” kit provided by Cayman (Cayman Chemical Co., Ann Arbor, MI, USA). The assay directly measures PGF2 a produced by SnCl_2_ reduction of COX-derived PGH2. The prostanoid production was quantified via enzyme immunoassay using a broadly specific antibody that binds to all the major prostaglandin compounds. The final estimation of % inhibition was performed at a substrate concentration much lower than the saturating concentration (0.1 μM, arachidonic acid). The compounds were added to the reaction mixture at a final concentration of 200μM. Naproxen used as positive controls, was added to the reaction mixture at the same concentration, 200μM, as the tested compounds [14].

### 3.4. Soybean Lipoxygenase Inhibition Study in Vitro

The compounds tested were dissolved in DMSO and preincubated for 4 min at 28 °C in the presence of 753 U/mL 5-soybean lipoxygenase (5-LOX). The enzyme reaction was triggered by addition of a final concentration of 0.1 mM sodium linoleate. The conversion of sodium linoleate 13-hydrouperoxylinoleic acid was measured at 234 nm. Absorbance was measured at time 0 and 3 min. Nordihydroguaretic acid (NDGA), an appropriate standard inhibitor, was used as positive control [14].

### 3.5. Molecular Docking Studies

Molecular docking studies were performed using X-ray crystal structures of COX-1 (PDB code: 1EQH) [60] and COX-2 (PDB code: 1PXX) [61] with bound inhibitors obtained from the Protein Data Bank (PDB) via AutoDock 4.2 program [62]. For the docking, the grid size was set to 50 × 50 × 50 xyz points with grid spacing of 0.375Å. For the preparation of ligand structures, 2D structure was sketched in chemdraw12.0, hydrogens were added and converted to mol2 format. For the docking simulation, default values of quaternation, translation and torsion steps were applied. The Lamarckian Genetic Algorithm with default parameters was applied for minimization. The number of docking runs was 100. The graphical depictions of all ligand-protein complexes were achieved by Discovery Studio Visualizer version 4.0 (BIOVIA, San Diego, CA, USA).

## 4. Conclusions

All twenty five substituted 5-benzylideno-2-adamantylthiazol[3,2-b][1,2,4]triazol-6(5*H*)ones showed high possibility to possess anti-inflammatory action according to PASS Program. The compounds were tested in vivo for anti-inflammatory activity and in vitro for COX-1/2 and LOX inhibitory action. All compounds exhibited in vivo anti-inflammatory action with eleven of them to possess equal or better activity than indomethacin. Among the halogen substituted compounds, the 2-Br (**17**), 3-Br (**18**) and 4-Cl (**22**) derivatives were the best with 43% to 30% increased activity compared to indomethacin. The 4-OMe derivative (**8**) exhibited activity comparable to that of the 4-Cl derivative (32% increase), while the 2-OH, 3-OMe derivative (**5**) showed 23% increased activity compared to the reference compound.

Most of the compounds exhibited low or no inhibition of COX-1/2 and LOX enzymes, indicating that the in vivo observed anti-inflammatory action is not due to the inhibition of COX/LOX pathways. As, several of these mechanisms are the main targets for specific inflammatory disorders such as migraine, these compounds are of great interest and worth further investigation. However, compound **8**, the compound with the highest anti-inflammatory action among the -OH/-OMe derivatives also exhibited remarkable, comparable to naproxen, COX-1 inhibitory action, while compound **3** showed extremely increased COX-1 inhibition with an IC_50_ at the micromolar rage, 30-fold lower than that of naproxen. COX-2 inhibitory action was observed in case of derivatives **2** (3-OH) and **6** (4-OH, 3-OMe) which was comparable to naproxen at high concentration but with IC_50_ higher than naproxen.

In conclusion, a number of compounds with good anti-inflammatory action were obtained. Although, some of them exhibited remarkable COX inhibitory action this activity did not follow the anti-inflammatory results, indicating the implication of other mechanisms, as well.

## Figures and Tables

**Table 1:**
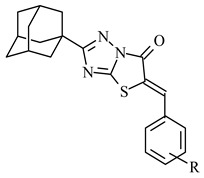
Anti-inflammatory activity (CPE% values) of substituted 5-benzyliden-2-adamantylthiazole [3,2-b] [1,2,4] triazol-6(5*H*)-one.

Com.	R	CPE, ^a^ %	Pa ^b^	Com	R	CPE ^a^ %	Pa ^b^
**1**	H	40	0.407	**14**	4-NO_2_	42	0.409
**2**	3-OH	36	0.551	**15**	3-F	51	0.581
**3**	4-OH	37	0.554	**16**	4-F	56	0.566
**4**	2,4-OH	49	0.480	**17**	2-Br	67	0.274
**5**	2-OH, 3-OMe	58	0.485	**18**	3-Br	61	0.360
**6**	4-OH-3-OMe	46	0.503	**19**	4-Br	53	0.393
**7**	4-OH, 3,5-OMe	36	0.567	**20**	2-Cl	47	0.585
**8**	4-OMe	62	0.539	**21**	3-Cl	45	0.636
**9**	3,4-OMe	44	0.538	**22**	4-Cl	61	0.552
**10**	3,4,5-OMe	49	0.503	**23**	2,3-Cl	37	0366
**11**	4-N(CH_3_)_2_	55	0.407	**24**	2,4-Cl	37	
**12**	2-NO_2_	40	0.409	**25**	2,6-Cl	32	0.585
**13**	3-NO_2_	15	-	**indomethacin**		47	

a-CPE—carrageenan paw edema; b—probability to be active.

**Table 2:** Cyclooxygenase (COX-1/-2) inhibitory effects in vitro.

A/A	R	COX-1 * (%)	COX-1 IC_50_ (μM)	COX-2 * (%)	COX-2 IC_50_ (μM)	LOX (%)
**2**	3-OH	18		64	>50	5.82
**3**	4-OH	93	1.28	25		28.58
**6**	4-OH 3 OCH_3_	26.2		66	>50	6.67
**7**	4-OH 3,5 di-OCH_3_	22		25		16.13
**8**	4-OCH_3_	60.5	41.88	0		5.72
**15**	3-F	36		23		27.15
**16**	4-F	19		0		16.13
**17**	2-Br	16		0		17.15
**18**	3-Br	0		37		31.43
**19**	4-Br	16		36		8.07
**23**	2,3 di-Cl	46		20		11.43
**Naproxen**	-	60	40.10	65	50	
**NDGA**	-	-	-	-		94

* at 200 μM.

**Table 3:** **COX1:** PDB1D: *1EQH* binding affinities.

Compound	R	Est. Binding Energy (kcal/mol)	Binding Affinity Dcore	I-H	Residues Involved at Hydrogen Bond Formation
**Flurbiprofen**		−8.8	−38.21	3	ARG120, TYR355
**3**	4-OH	−8.2	−31.67	2	ARG120, TYR355
**8**	4-OMe	−7.0	−23.49	1	TYR355
**18**	3-Br	−4.4	−14.88	1	SER530

**Table 4:** **COX2:** PDBID: *1PXX* binding affinities.

Compound	R	Est. Binding Energy (kcal/mol)	Binding Affinity Dcore	I-H	Residues Involved at Hydrogen Bond Formation
**diclofenac**		−9.0	−32.95	3	Tyr385, SER530
**2**	3-OH	−8.8	−29.99	2	TYR355, ARG120
**6**	4-OH,3-OMe	−8.6	−30.82	2	TYR355, ARG120
**16**	4-F	−0.9	−5.78	0	-
**Naproxen**		−8.0	−28.67	1	ARG120
